# A prospective international multi-center study on safety and efficacy of deep brain stimulation for resistant obsessive-compulsive disorder

**DOI:** 10.1038/s41380-019-0562-6

**Published:** 2019-10-29

**Authors:** José M. Menchón, Eva Real, Pino Alonso, Marco Alberto Aparicio, Cinto Segalas, Gerard Plans, Laura Luyten, Els Brunfaut, Laurean Matthijs, Simon Raymakers, Chris Bervoets, Antonio Higueras, Majed Katati, José Guerrero, Mariena Hurtado, Mercedes Prieto, Lennart H. Stieglitz, Georg Löffelholz, Sebastian Walther, Claudio Pollo, Bartosz Zurowski, Volker Tronnier, Andreas Kordon, Orsola Gambini, Rebecca Ranieri, Angelo Franzini, Giuseppe Messina, Diana Radu-Djurfeldt, Gaston Schechtmann, Long-Long Chen, Renana Eitan, Zvi Israel, Hagai Bergman, Tim Brelje, Thomas C. Brionne, Aurélie Conseil, Frans Gielen, Michael Schuepbach, Bart Nuttin, Loes Gabriëls

**Affiliations:** 1 Bellvitge University Hospital-IDIBELL, University of Barcelona, CIBERSAM, Barcelona, Spain; 2 KU Leuven and/or UZ Leuven and/or UPC KU Leuven, Leuven, Belgium; 3 Hospital Virgen de las Nieves, Granada, Spain; 4 Inselspital Bern, Bern, Switzerland; 5 Translational Research Center, University Hospital of Psychiatry, Bern, Switzerland; 6 Universitätsklinik Schleswig-Holstein, Campus Lübeck, Lübeck, Germany; 7 Oberbergklinik Schwarzwald, Hornberg, and Universitätsklinikum Freiburg, Klinik für Psychiatrie und Psychotherapie, Freiburg, Germany; 8 Department of Health Sciences, University of Milano, San Paolo Hospital Milano, Milano, Italy; 9 Fondazione IRCCS Istituto Naz Neurologico C.Besta, Milano, Italy; 10 Psykiatri Sydvast, OCD-departement, Karolinska University Hospital-region in Huddinge, Stockholm, Sweden; 11 Department of Neurosurgery, Karolinska Institutet and University Hospital, Stockholm, Sweden; 12 Psychiatry Department, Hadassah-University Hospital, Jerusalem, Israel; 13 Medtronic, Minneapolis, USA; 14 Medtronic International Trading Sàrl, Tolochenaz, Switzerland; 15 Medtronic Bakken Research Center, Maastricht, The Netherlands

**Keywords:** Psychiatric disorders, Depression

## Abstract

Deep brain stimulation (DBS) has been proposed for severe, chronic, treatment-refractory obsessive-compulsive disorder (OCD) patients. Although serious adverse events can occur, only a few studies report on the safety profile of DBS for psychiatric disorders. In a prospective, open-label, interventional multi-center study, we examined the safety and efficacy of electrical stimulation in 30 patients with DBS electrodes bilaterally implanted in the anterior limb of the internal capsule. Safety, efficacy, and functionality assessments were performed at 3, 6, and 12 months post implant. An independent Clinical Events Committee classified and coded all adverse events (AEs) according to EN ISO14155:2011. All patients experienced AEs (195 in total), with the majority of these being mild (52% of all AEs) or moderate (37%). Median time to resolution was 22 days for all AEs and the etiology with the highest AE incidence was ‘programming/stimulation’ (in 26 patients), followed by ‘New illness, injury, condition’ (13 patients) and ‘pre-existing condition, worsening or exacerbation’ (11 patients). Sixteen patients reported a total of 36 serious AEs (eight of them in one single patient), mainly transient anxiety and affective symptoms worsening (20 SAEs). Regarding efficacy measures, Y-BOCS reduction was 42% at 12 months and the responder rate was 60%. Improvements in GAF, CGI, and EuroQol-5D index scores were also observed. In sum, although some severe AEs occurred, most AEs were mild or moderate, transient and related to programming/stimulation and tended to resolve by adjustment of stimulation. In a severely treatment-resistant population, this open-label study supports that the potential benefits outweigh the potential risks of DBS.

## Introduction

Obsessive-compulsive disorder (OCD) is a chronic and disabling neuropsychiatric disorder characterized by the presence of obsessions—upsetting and repetitive thoughts, images, or impulses, experienced as intrusive, that persist despite efforts to suppress, resist, or ignore them, and/or compulsions, repetitive, ritualized behaviors or mental acts intended to neutralize the anxiety induced by the obsessions [1]. OCD has a lifetime prevalence of 2.3% [2] and can cause profound, life-impairing stress and substantial dysfunction in social, work, and family life [3]. According to the World Health Organization, OCD is among the top 20 causes of illness-related disability for people aged 15–44 years [4]. Despite exhaustive use of optimal cognitive-behavioral therapy and pharmacological treatment algorithms, an estimated 10% of OCD patients remain unresponsive to all therapies and suffer from severe symptoms leading to marked functional impairment [5].

For this group of extremely disabled patients, deep brain stimulation (DBS) has been recently approved as an alternative to stereotactic ablative neurosurgery. Medtronic Reclaim™ DBS Therapy for OCD received a humanitarian device exemption from the FDA and received a CE mark for its use in intractable severe OCD in 2009. ‘Approved’ thus refers to this regulatory approval rather than the subsequently published consensus on guidelines from physician societies [6]. Although DBS is an invasive procedure with potentially serious adverse events (SAEs), the technique has several advantages: it is reversible, it allows for optimization of parameters—selection of active electrode contacts, frequency, polarity, and intensity—for individual patients, provides opportunities to study the brain circuitry involved, and enables sham treatment studies that may help to obtain stronger evidence for surgical therapies.

Several DBS targets have been tested in OCD, including structures around the ventral capsule/ventral striatum (VC/VS), the subthalamic nucleus (STN) and the inferior thalamic peduncle [7]. The approved indication prescribes stimulation of the anterior limb of the internal capsule (AIC), which could be achieved with an active electrode located in AIC itself, the VC/VS interface, the neighboring nucleus accumbens or the bed nucleus of the stria terminalis (BST) [8].

To date, seven studies that included randomized, controlled assessments of DBS in 69 OCD patients have been published. However, most of the literature on effectiveness of DBS consists of uncontrolled case reports, series or trials [8–14]. Recent systematic reviews and meta-analyses estimate reduction in OCD severity in response to DBS to be 45–48%. The global percentage of responders—patients with at least a 35% reduction in Yale-Brown Obsessive-Compulsive Scale (Y-BOCS) scores—reaches 58–67% [7, 8, 15, 16].

Relatively little is known on the detailed safety profile of DBS for psychiatric disorders in general, and specifically for OCD. A recent review suggests that adverse events (AEs) of DBS in OCD, Gilles de La Tourette’s syndrome and treatment-resistant depression are similar to those reported for DBS use in movement disorders, with the majority of complications being transient and related to stimulation [17]. Nevertheless, long-term morbidity occurred in 16.5% of the cases and permanent neurological complications due to intracerebral hemorrhage (ICH) were reported in 2.2% of the patients [17]. To allow an adequate assessment of the benefit-risk ratio of DBS for OCD, further studies with a rigorous report of positive as well as negative outcomes and AEs are mandatory.

This study constitutes the first prospective, open-label, interventional multi-center study aimed to monitor the safety and performance of electrical stimulation of the AIC in patients with chronic, severe, treatment-resistant OCD (ClinicalTrial.gov identifier: NCT01135745**)**. It was sponsored by Medtronic, as part of the postmark clinical follow-up commitment made to the notified body that granted CE mark for the therapy in Europe. The objectives of this study were (1) to characterize AEs associated with the implant procedure, the device and the bilateral stimulation of the AIC in OCD patients and (2) to assess the efficacy of DBS treatment on OCD symptoms, quantified with the Y-BOCS, from baseline to 3, 6, and 12 months after implantation.

## Materials and methods

For detailed ‘Materials and methods”, refer to Supplementary Appendix.

Patients suffered from severe to extreme OCD according to the Y-BOCS (total score of at least 30/40) [18], and were seriously impaired in daily functioning. This level of impairment had persisted for ≥5 years despite a minimum of three adequate pharmacological trials with first- and/or second-line medications (at least one trial had to be with clomipramine) and a supplementary augmentation trial (atypical neuroleptics). Included patients had not responded to an adequate trial of cognitive-behavioral therapy. Patients were excluded if they had a current Axis I disorder that was primary to OCD according to the Structured Clinical Interview for DSM-IV-TR Axis I Disorders (SCID-I) [19], if they met criteria for substance abuse or dependence ≤6 months prior to the screening test, if they made suicide attempts ≤3 months prior to screening (or posed a serious suicide risk) or if they had any neurological condition that could hinder the stimulation procedure (more details on inclusion/exclusion criteria are shown in Supplementary Table 1 and a diagram of inclusion is shown in Supplementary Fig. 1).

After a preoperative MRI, model 3391 DBS leads [Medtronic, Inc., Minneapolis, MN, USA] were stereotactically implanted in the bilateral AIC and connected subcutaneously to unilateral or bilateral dual-channel Model 7428 Kinetra™, bilateral single-channel Model 7426 Soletra™ or unilateral dual-channel Model 37601 Activa™ PC neurostimulators [Medtronic, Inc.]. Activa™ PC and the single-channel Model 37602 and 37603 Activa™ SC neurostimulators have since received CE mark for OCD. Implanted 3391 electrodes feature 3-mm-long contacts 4 mm apart, spanning a total length of 24 mm. The objective of electrode location was to enable stimulation of the AIC, but did not impose a detailed targeting technique. Each neurosurgeon chose surgical trajectory and lead end point, based on clinical expertize and individual anatomical characteristics of the patient. A commercialized image-guided software system was used for all lead implantations. Postoperative imaging (CT and/or MRI) was performed to document lead location. Following surgical recovery, patients underwent DBS parameter selection to identify optimal parameter settings for the treatment phase (within 4 weeks of implantation). During this treatment phase, safety and efficacy assessments were performed at 3, 6, and 12 months post implant (study flow is shown in Supplementary Fig. 2 and study timeline is shown in Supplementary Fig. 3). Stimulation parameters and psychotropic medication could be modified in response to AEs and/or in order to improve efficacy as necessary. All AEs and SAEs had to be followed up in accordance with good medical practice until resolved or judged no longer clinically significant. An independent Clinical Events Committee reviewed all AEs. The definition of AEs and SAEs is provided in Supplementary Appendix.

To characterize DBS efficacy, the following measurements were taken: the Y-BOCS (for assessing the severity of obsessive-compulsive symptoms), the Global Assessment of Functioning (GAF) [20, 21] for measuring psychological, social and occupational functioning, the Montgomery-Åsberg Depression Rating Scale [22] for assessing the severity of depressive symptoms, the Young Mania Rating Scale (YMRS) [23] for measuring the severity of manic symptoms, the Clinical Global Impressions Scale (CGI) [24] for rating the severity of psychiatric illness and symptom improvement, and the EuroQol group-5 Dimensional (EQ-5D) as a measure of health outcome [25].

## Results

### Patients

Demographic data and OCD characteristics are summarized in Supplementary Table 2. Thirty-one patients were enrolled and 30 patients implanted across eight centers in Europe and Israel. One additional patient was included but asked to retract his data after finishing the study and has not been included in this report. Half the patients were male (15, 48%) and mean (standard deviation [SD]) age at enrollment was 41.0 (9.9) years. The vast majority exhibited a high impairment in social and occupational functioning, with mean (SD) GAF score at baseline 39.0 (8.1). Mean baseline Y-BOCS total score was 34.7 (2.9), indicating extreme OCD. Current and lifetime prevalence of psychiatric comorbidities were assessed through SCID-I. The most common lifetime comorbidities were mood disorders (19 patients, 61%), anxiety disorders (other than OCD) (12, 39%) and substance use disorders (7, 23%), and the most common current comorbidities were mood disorders (9, 29%), anxiety disorders (9, 29%) and somatoform disorders (5, 16%). Almost half of the patients (15, 48%) did not report any current psychiatric comorbidity at screening.

### Implant procedure and therapy delivery

**Fig. 1:**
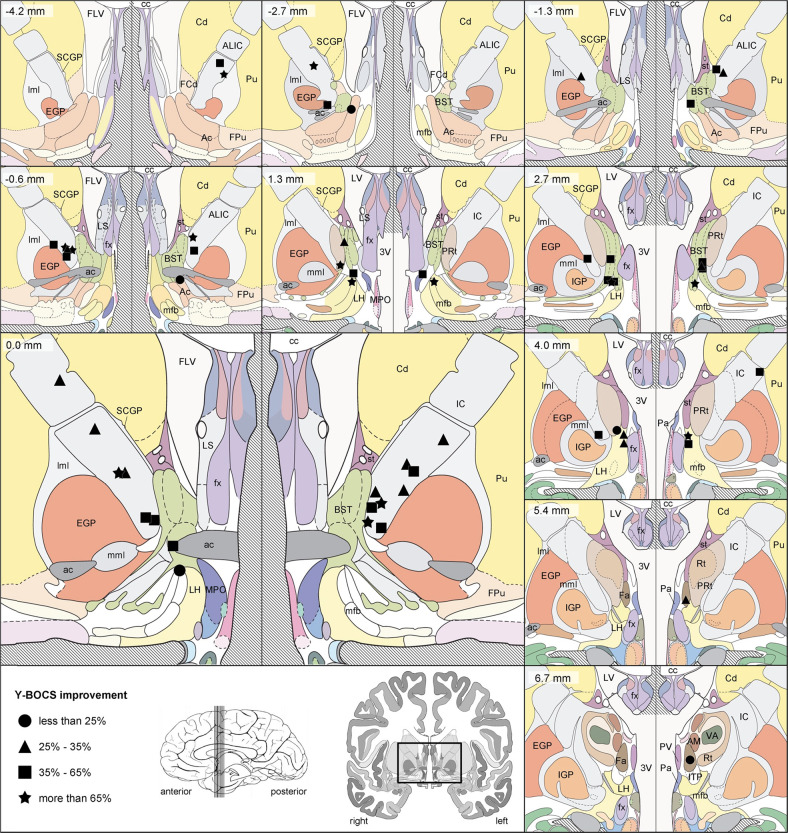
Coronal brain atlas slices showing the location of the center of all 58 cathodes at 12 months follow-up (26 patients). Contacts are depicted with different symbols, according to the patient’s Y-BOCS improvement at 12 months follow-up (versus preoperative baseline): ● indicates less than 25% improvement, ▲ 25–35%, ■ 35–65% and ⋆ more than 65% improvement. One subject was excluded because, although the neurostimulator was on at Month 12, it was unclear for what actual duration the therapy had been delivered, due to low impedance. Note that three patients were stimulated with two or three contacts per hemisphere, whereas one patient was only stimulated with one active contact on the right side. All 22 other patients were stimulated bilaterally, with one cathode in each hemisphere. In the top left corner of each slice, the position anterior (−) or posterior (+) to the anterior commissure is specified. For each coronal slice, the right and left hemispheres are depicted adjacently, in accordance with the radiological convention (right hemisphere shown on the left side and vice versa). Instead of displaying complete coronal slices, a detailed window is shown, dorsally bordered by the corpus callosum and ventrally extending 10 mm below the intercommissural plane, as indicated in the bottom left panel. In addition, a sagittal view of the brain with indication of the relevant coronal slices is shown. ac anterior commissure, Ac nucleus accumbens, AIC anterior limb of the internal capsule, AM anteromedial thalamic nucleus, BST bed nucleus of the stria terminalis, cc corpus callosum, Cd caudate nucleus, EGP external globus pallidus, Fa fasciculosus nucleus, FCd fundus region of caudate nucleus, FLV frontal horn of lateral ventricle, FPu fundus region of putamen, fx fornix, IC internal capsule, IGP internal globus pallidus, ITP inferior thalamic peduncle, LH lateral hypothalamic area, lml lateral medullary lamina of globus pallidus, LS lateral septal nucleus; LV lateral ventricle, mfb medial forebrain bundle, mml medial medullary lamina of globus pallidus, MPO medial preoptic nucleus, Pa paraventricular hypothalamic nucleus, PRt prereticular zone, Pu putamen, PV paraventricular thalamic nucleus, Rt reticular thalamic nucleus, SCGP supracapsular part of globus pallidus, st stria terminalis, VA ventral anterior thalamic nucleus, 3V third ventricle. Images are adapted from Mai’s Atlas of the Human Brain [43]

Several neurostimulators are CE marked for OCD, and the neurostimulator model used in the majority of 30 implanted patients was Kinetra™ (27, 90%). Of these patients, 19 (63% of all implanted) had one neurostimulator and a further 8 (27%) had two neurostimulators implanted. Of the remaining 3 patients, 2 (7%) had Activa™ PC neurostimulators (one for each patient) and 1 patient had two Soletra™ neurostimulators. DBS stimulation was initiated in all 30 implanted patients. At the Month 12 visit, 27 (90%) patients still had active devices, although 1 was not receiving effective stimulation due to low impedance. Of the 26 patients receiving effective stimulation, 25 received bilateral stimulation as intended per approved indication and 1 received unilateral stimulation. Leads were programmed with 1 active contact, except for 5 leads with 2 active contacts, and 1 lead with 3 active contacts, totaling 58 active contacts at Month 12. The anatomical location of the geometric center of active contacts at Month 12 is shown in Fig. 1.

At the last available visit that patients received stimulation (including the 1 patient with low impedance at Month 12, and adding 1 patient with bilateral stimulation at an earlier visit), 55 hemispheres were stimulated. Lead location varied according to individual patient anatomy and the neurosurgeon’s clinical decision. Stimulation settings, including contact selection, were based on psychiatrist assessment of response to test stimulation, and adjusted when necessary over the entire follow-up period. Anatomical location of the active contacts therefore varied according to both initial implant and programming technique over the course of the study. The most common location for the center of the active contact was the anterior internal capsule (ventral part of AIC, in 31 hemispheres, 56% of the 55 stimulated hemispheres), followed by the bed nucleus of the stria terminalis (15 hemispheres, 27%), lateral hypothalamus (3 hemispheres, 5%), globus pallidus externus (2 hemispheres) and dorsal part of AIC (1 hemisphere). As mentioned above, some patients had leads with more than one active contact, and in three of these patients, the centers of the active contacts were located in adjacent brain areas. In one hemisphere, this was in the ventral part of AIC plus bed nucleus of the stria terminalis, in another one in the ventral part of AIC plus lateral hypothalamus, and in a third hemisphere this was in the ventral part of AIC plus globus pallidus externus plus dorsal part of AIC. Note that the actual stimulated anatomical structures probably extended beyond the center of each active contact displayed in Fig. 1 [8]. Of the three patients with their stimulator turned off at Month 12, two patients had the device explanted (in one case due to intracranial infection and in the other case due to a device event: extension migration/dislodgement). The third patient had a temporary suspension of therapy due to possible magnetic field action which may have inadvertently shut off the stimulation.

Stimulation settings were rather high compared to DBS for movement disorders (mean [SD] at Month 12: amplitude 4.7 (1.8) Volts, pulse duration 221 (63) µs, frequency 130 (3) Hz) but were in line with previous literature on DBS for OCD [8, 26]. A total of eight battery replacements were made for end of battery life during the study (all of them were made between 5 and 10 months after the original implant).

### Safety evaluation

#### Adverse events

An overview of AEs that occurred during the study is presented in Table 1. All patients experienced AEs (195 in total), with the majority of them being mild (*n* **=** 102, corresponding to 52% of AEs) or moderate (73, 37%). Out of the 195 AEs, 123 (63%) were device-related, i.e. could be attributed to the device, the procedure or the electrical stimulation. Half of the patients reported between one and five AEs each, with the remainder reporting six AEs or more (3 patients reported > 10 AEs). Median time to resolution was 22 days for all AEs.

**Table 1:** Nonserious adverse events by high level group term reported in >1 patient

MedDRA high level group term	N Pts	N AEs	Number of AEs with etiology										Diagnoses (N AEs)
			Preexisting condition	New illness	Medication	Surgery/anesthesia	Incisional site/device tract	Device	Programming/ stimulation: possible	Programming/ stimulation: probable	Programming/ stimulation: definite	Other, unknown	
Headaches	11	12	3				3	1	1			4	Headache (12)
Neurological disorders NEC	10	17		2	2		3		5	1	3	1	Sensory disturbance (3), Paresthesia (3), Dizziness (2), Dysarthria (2), Balance disorder (1), Dysesthesia (1), Dysgeusia (1), Neuralgia (1), Restless legs syndrome (1), Sedation (1), Syncope (1)
Sleep disorders and disturbances	9	10							6	3	1		Insomnia (7), Initial insomnia (2), Sleep disorder (1)
Anxiety disorders and symptoms	8	11	2		1	1			1	2	4		Anxiety (3), Worsening OCD (3), Nervousness (2), Tension (2), Panic attack (1)
Administration site reactions	8	8				1	5	2					Implant site pain (7), Implant site irritation (1)
Infections-pathogen class unspecified	6	9		8		1							Nasopharyngitis (5), Bronchitis (1), Impetigo (1), Pharyngitis (1), Sinusitis (1)
General system disorders NEC	6	7	1			2			2	2			Asthenia (2), Chest pain (2), Fatigue (1), Feeling abnormal (1), Pain (1)
Mood disorders and disturbances NEC	6	6							4	1	1		Inappropriate affect (2), Affect lability (1), Affective disorder (1), Apathy (1), Mood altered (1)
Epidermal and dermal conditions	5	5	1		2	1						1	Pruritus generalized (3), Dermatitis allergic (1), Scar pain (1)
Procedural and device-related injuries and complications NEC	5	5					3	2					Medical device discomfort (2), Incision site complication (1), Seroma (1), Wound dehiscence (1)
Depressed mood disorders and disturbances	4	5	1					1	1	2			Depression (4), Depressed mood (1)
Musculoskeletal and connective tissue disorders NEC	3	4				1		1	2				Musculoskeletal discomfort (3), Shoulder pain (1)
Respiratory disorders NEC	3	4		3		1							Cough (3), Pharyngolaryngeal pain (1)
Movement disorders (incl Parkinsonism)	3	3			1					1		1	Dyskinesia (1), Psychomotor hyperactivity (1), Tremor (1)
Psychiatric and behavioral symptoms NEC	3	3							2	1			Abnormal behavior (3)
Urinary tract signs and symptoms	3	3	2								1		Urinary incontinence (3)
Gastrointestinal signs and symptoms	2	3				1				1		1	Abdominal pain (1), Nausea (1), Vomiting (1)
Deliria (incl confusion)	2	2							1	1			Delirium (1), Disorientation (1)
Gastrointestinal motility and defecation conditions	2	2	1	1									Constipation (1), Diarrhea (1)
Mental impairment disorders	2	2	1									1	Cognitive disorder (1), Memory impairment (1)
Muscle disorders	2	2		1								1	Muscle spasms (1), Myalgia (1)
Personality disorders and disturbances in behavior	2	2							1	1			Irritability (1), Paranoia (1)
Physical examination topics	2	2							1			1	Weight decreased (1), Weight increased (1)
Thyroid gland disorders	2	2	1	1									Hypothyroidism (2)
Total^a^	29	129	13	16	6	9	14	7	27	16	10	11	

NEC not elsewhere classified, AE adverse event

^a^Total number of AEs equals 129 (instead of 195) because only nonserious AEs happening in >1 patient for the HLGT (High Level Group Term) were displayed

Totaling serious and nonserious AEs, the Medical Dictionary for Regulatory Affairs (MedDRA) High Level Group Term (HLGT) with the highest incidence of AEs was Anxiety disorders and symptoms, followed by Neurological disorders not elsewhere classified, and Headaches. According to the Clinical Events Committee classification, the etiology with the highest AE incidence was ‘programming/stimulation’, with events reported in 26 patients (84% of enrolled), followed by ‘New illness, injury, condition’ (13, 42%), ‘preexisting condition, worsening or exacerbation’ (11, 35%), ‘incisional site/device tract’ (11, 35%), ‘Medication’ (10, 32%) and ‘Unknown’ (10, 32%).

#### Serious adverse events

A total of 36 SAEs was reported by 16 patients (52%) (Table 2). The most frequently reported SAE was OCD worsening (10 events in 9 patients, 29%), followed by seizures (5 events in 4 patients, 13%), anxiety and hypomania (2 events in 2 patients each, 6%). Some investigators considered ‘OCD worsening’ as referring to the preceding visit, others to the baseline visit. Two generalized tonic-clonic seizures occurred in a patient in whom an electrode was misplaced in the dorsal caudate nucleus. One patient presented a similar episode 48 h after electrode implant accompanied by a confusional state. In one patient a seizure occurred in the context of an intracranial infection. In one patient a single seizure episode took place 6 months after implantation while modifying stimulation parameters. It is noteworthy that 8 of the 36 SAEs were reported in one single patient (convulsion, coma, infection, pleural effusion, axillary vein thrombosis, bronchopneumonia, pneumothorax, and shock), all attributed to surgery-related infection. This patient’s DBS system was explanted 5.4 months after implantation (the patient remained in the study, but without DBS) and almost all events resolved without sequelae; the exceptions were pleural effusion (resulted in dysphonia) and bronchopneumonia (resulted in modest dysventilation on the chest X-ray). Overall median time to resolution was 27 days for SAEs.

**Table 2:** Serious adverse events by high level group term

MedDRA high level group term	N Pts	N SAEs	Number of SAEs with etiology								Diagnoses (N SAEs)
			Preexisting condition	New Illness	Medication decrease	Surgery/ anesthesia	Device	Programming/ stimulation: possible	Programming/ stimulation: probable	Other, unknown	
Anxiety disorders and symptoms	9	12			3	2	2	1	2	2	OCD symptoms (10), Anxiety (2)
Seizures (incl subtypes)	4	5				2			1	2	Generalized tonic-clonic (3 + 1^a^), Generalized tonic (1)
Manic and bipolar mood disorders and disturbances	2	2							2		Hypomania (2)
Suicidal and self-injurious behaviors NEC	2	2	1						1		Suicidal ideation (1), Suicidal attempt (1)
Infections - pathogen class unspecified	1	2				2					Bronchopneumonia (1^a^), Intracranial Infection (1^a^)
Lifestyle issues	1	2	1					1			Benzodiazepine abuse (1)/overdose (1)
Pleural disorders	1	2				2					Pleural effusion (1^a^), Pneumothorax (1^a^)
Decreased and nonspecific blood pressure disorders and shock	1	1				1					Shock (1^a^)
Deliria (incl confusion)	1	1				1					Confusional state (1)
Dissociative disorders	1	1							1		Dissociation (1)
Embolism and thrombosis	1	1				1					Axillary vein thrombosis (1^a^)
Family issues	1	1	1								Marital problem (1)
Mood disorders and disturbances NEC	1	1						1			Dysphoria (1)
Neurological disorders NEC	1	1				1					Pharmacologically induced coma (1^a^)
Personality disorders and disturbances in behavior	1	1	1								Borderline personality disorder (1)
Thyroid gland disorders	1	1		1							Hypothyroidism (1)
Total	16	36	4	1	3	12	2	3	7	4	

There were no serious adverse events with etiology of programming/stimulation: definite or incisional site/device tract

*NEC* not elsewhere classified, *SAE* serious adverse event

^a^One patient experienced these eight SAEs

None of the AEs led to premature discontinuation of the study, but AEs led to 12 temporary therapy suspensions in 8 patients for several reasons (seizures, headache, chest pain, restlessness, hypomania, hypersomnia, and OCD). The vast majority of AEs was resolved at the end of the study and only 9 events (5% of total AEs) in 7 patients were ongoing at the end of the follow-up period (this included two patients with three SAEs, one with an SAE of hypothyroidism and another patient with SAEs of worsening emotional instability within borderline personality disorder and suicidal ideation). The six ongoing nonserious AEs were dizziness, hypercholesterolemia, gastritis, implant site pain (one subject each), and one subject with both hypothyroidism and tremor.

#### Acute stimulation-induced effects (ASIEs)

ASIEs were not included in the definition of an AE, but provided valuable information on DBS treatment. By definition, these effects were transient and resolved with or without programming changes prior to the patient leaving a study visit. A total of 588 ASIEs were reported at 34% of visits at which stimulation parameters were adjusted. The most common ASIEs experienced at the first parameter selection visit were increased anxiety (10 patients, 33% of stimulated), sensations of cold or hot (9, 30%), other mood and anxiety effects (8, 27%), and skin (facial) flushing (7, 23%).

### Efficacy

**Fig. 2:**
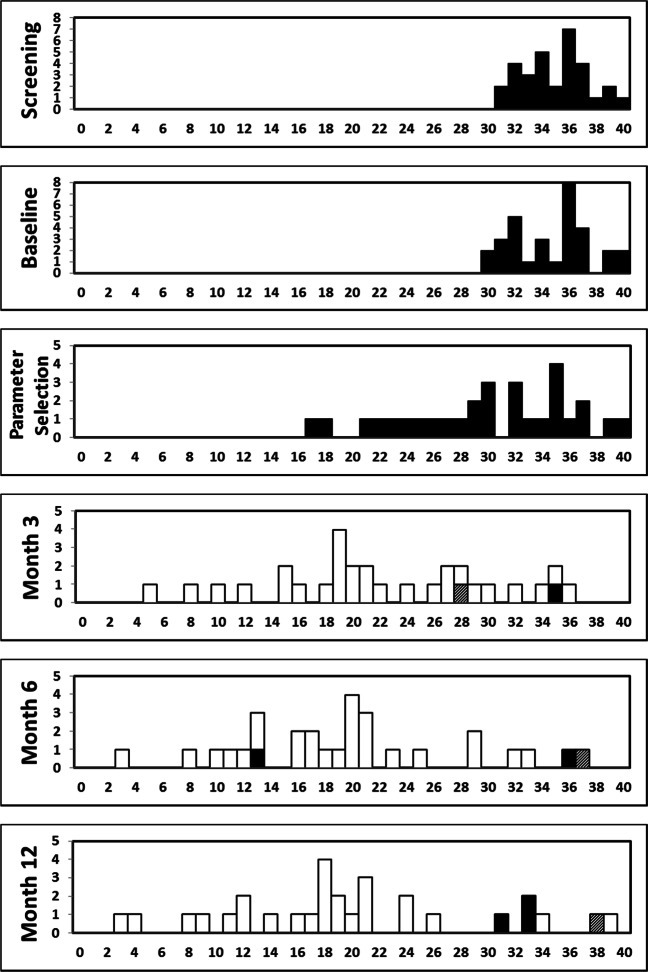
Frequency distribution of the individual patients’ Y-BOCS Total scores at each visit. Total *n* **=** 31 enrolled patients. Stimulation status: white = on bilateral, black = off, hatched = on unilateral. *X*-axis: Y-BOCS total score; *Y*-axis: count of patients

Frequency distributions of Y-BOCS total score at each visit are shown in Fig. 2. The mean (SD) Y-BOCS total score for the implanted population decreased from baseline to the first Parameter Selection visit (the Y-BOCS was assessed at this visit prior to any stimulation) from 34.9 (2.9) to 30.0 (6.1), corresponding to a mean (SD) reduction from baseline of 14% (15%). Following initiation of stimulation, a further decrease in the mean Y-BOCS was observed during the treatment period, with an improvement observed as early as Month 3. The mean (SD) score was 22.3 (8.2) at Month 3, 19.8 (8.3) at Month 6, and 20.0 (9.5) at Month 12, corresponding to a mean (SD) reduction from baseline of 36% (23%), 43% (23%), and 42% (27%), respectively. The majority of patients (17 of 30, 57%) could be classified as Y-BOCS responders (at least a 35% reduction in Y-BOCS score from baseline) at Month 3. The number of responders at Month 6 and Month 12 was 21 patients (70%) and 18 patients (60%), respectively. Among responders at Month 12, most patients had active contacts located bilaterally within AIC (6 patients, 33%) or BST (6 patients, 33%). Among nonresponders, most patients had active contacts located bilaterally within AIC (4 patients, 33%) or patients were not receiving stimulation (3 patients, 25%). Mean scores for obsessions and compulsions decreased with a similar magnitude to 10.0 (5.1) and 10.0 (4.6) at Month 12 for Y-BOCS obsessions and compulsions, respectively.

**Fig. 3:**
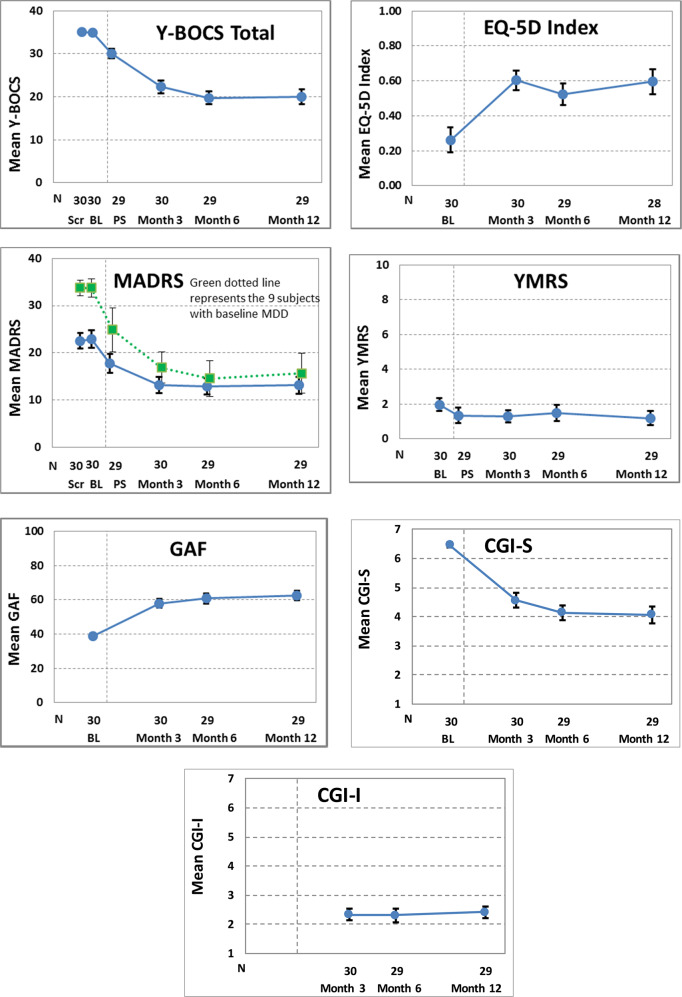
Efficacy measurements’ changes across visits. *n* **=** 30 implanted patients. The blue circles and solid lines represent all patients. On the MADRS plot, the green squares and dotted line represent the 9 patients with baseline MDD. Scr screening visit, BL baseline visit, PS parameter selection visit. Error bars represent ± 1 standard error. Y-BOCS Yale–Brown Obsessive-Compulsive Scale, MADRS Montgomery-Åsberg Depression Rating Scale, GAF Global Assessment of Functioning, EQ-5D EuroQol group-5 Dimensional, YMRS Young Mania Rating Scale, CGI-I Clinician Global Impressions of Improvement, CGI-S Clinician Global Impressions of Severity

Changes in additional efficacy measures supported the observed effects on Y-BOCS, with mean values for most measures (GAF, MADRS, CGI, and EQ-5D) showing improvement, and the YMRS maintaining a low level, over the 12 months of follow-up (Fig. 3). Note that, analogous to the Y-BOCS total score, the mean MADRS scores also decreased in those 9 patients suffering from comorbid major depressive disorder.

## Discussion

This is the first prospective, international multi-center study designed to monitor the safety and efficacy of DBS of the AIC for patients with chronic, severe, treatment-resistant OCD. Although AEs were frequent, the majority were mild or moderate, transient, and mostly related to programming and could then be resolved by adjusting stimulation parameters. Only nine AEs (5% of the total) persisted at the end of the study, while the majority resolved within an average of three weeks. After 12 months of stimulation, the mean Y-BOCS score was decreased by 42%, and 60% of the patients could be considered responders. Comorbid depressive symptoms, global functioning and health level all improved in response to DBS.

A recent review on the safety profile of DBS use in psychiatric disorders concluded that the majority of reported complications for these indications are mild to moderate, stimulation-related, and transitory as many resolved promptly with modification of stimulation parameters [17]. For OCD patients, mood changes, including both depressive and manic states, as well as anxiety symptoms going from transient OCD worsening to inner tension or restlessness are the most frequently reported AEs associated with DBS use [15, 17]. Ventral stimulation of the AIC-nucleus accumbens has been described to be associated with the emergence of fear and panic attacks in OCD patients [27] as well as with apathy and depression in patients with Gilles de La Tourette syndrome [28]. Hypomania was frequently observed in OCD patients treated by STN DBS. [13] Accordingly, in our study, OCD, seizures, anxiety, and hypomania were recorded as the most frequent SAEs. Worsening of OCD symptoms was the most common adverse event recorded per protocol (and considered ‘serious’ by definition) in our study. However, although fluctuations in symptom severity were common during adjustment of stimulation parameters, they were always transient and reversible.

Four patients in our study, 13% of the sample, presented with seizures, mostly generalized tonic-clonic seizure episodes. Seizures have been described as an uncommon complication of DBS surgery in patients with movement disorders, with percentages ranging from 4.3% to 6.4% of the samples [29, 30] and have been reported previously in DBS patients with OCD [8, 26]. They generally occur within 48 h of surgery and are especially associated with the presence of intracranial vascular events including hemorrhage, edema, or ischemia [29].

While seizures are an expected potential side effect of DBS therapy, this higher than expected number may have come from a combination of factors in our sample. Unlike in the other studies on DBS for OCD, one subject experienced an intracranial infection leading to a seizure. Another subject experienced two generalized tonic-clonic seizures when receiving high frequency stimulation through a misplaced electrode in the caudate nucleus. In a few animal models and human studies, low frequency stimulation of the caudate nucleus (<50 Hz) could reduce epileptic activity, while high frequency stimulation of the caudate nucleus (i.e. in ranges similar to those used in our study) could increase epileptic activity [31]. A similar mechanism may have caused these two seizures. The remaining two subjects with seizures are within the range that was reported in prior DBS for OCD studies [8, 26].

Suicide risk associated with DBS use in OCD was low in our study, with one patient reporting suicidal ideation and one performing a suicide attempt. Suicidality has been described as the most frequent SAE of DBS in psychiatric disorders, especially in patients with Major Depressive Disorder (MDD) [17]. According to a recent meta-analysis, 3.4% of OCD patients who received DBS reported suicidal ideation [15]. Suicidality is a complex multi-factorial phenomenon and published studies have not been able to establish a definitive causal relationship between DBS surgery and suicidal ideation or attempts [32, 33]. Postoperative depression, being single, and a previous history of impulse control-related behaviors emerged as independent risk factors in case series of parkinsonian patients [34, 35]. The presence of unrealistic expectations about surgical outcome has also been suggested to play a significant role in suicidal ideation.

Surgery- and device-related complications were limited, with implant site pain and irritation as the most frequent ones. Nevertheless, one patient had an intracranial infection that forced an explant of the DBS system and led to permanent sequelae in the form of dysphonia and modest pulmonary dysventilation. ICH, one of the most severe risks of DBS, did not occur in our sample. ICH in OCD patients implanted for DBS has been reported to arise in 2.2–2.6% of the cases [7, 8, 15], a percentage somewhat similar to the rate of ICH in DBS for movement disorders, estimated to be between 0.8 and 3.3% [36, 37].

Sixty percent of the patients qualified as treatment responders. The mean reduction on Y-BOCS scores was over 40%. These results are almost identical to those reported in a meta-analysis of DBS use in OCD patients [15] and confirm what can be expected from the technique concerning efficacy. Interestingly, a decrease of 14% on Y-BOCS scores was detected immediately after the device implant and before initiating stimulation, an effect that may be attributed to placebo response, regression to the mean, or to the edema inflicted by the device implantation and that was largely known to occur in lesional procedures [38]. Response to DBS in OCD patients seems to occur relatively early, with a clear and significant amelioration of symptom severity after just three months of active stimulation.

Regrettably, 40% of the patients in our sample did not reach the responder criterion at the Month 12 visit, although 5 of these 12 ‘nonresponders’ did reach partial response (25–35% reduction vs. baseline Y-BOCS score). In the case of OCD, the therapeutic effect of DBS has been tentatively related to its capacity to modulate abnormal activity and synaptic connectivity in circuits involving the orbitofrontal cortex (OFC), anterior cingulate cortex (ACC) and striatum [16, 39], circuits that have been implicated in the pathophysiology of the disorder [40]. Individual anatomical variability of the orbitofrontothalamic fibers in the stimulated region might partially explain differences in the response rate. In addition, variations in the final locations of the active electrode contacts could also be related to response rates. Recently developed tractographic techniques allow measuring this individual variability, and thus might permit targeting individualized anatomical structures for maximum response to DBS [41].

Limitations of the study: This is a nonrandomized study, based on nonblinded assessments, and therefore influence of placebo effect on efficacy could not be ruled out. However, randomized studies in OCD patients comparing active versus sham DBS have consistently reported severe clinical deterioration associated with sham conditions, suggesting that a placebo effect, quite limited in OCD patients in general, is almost absent in this group of extremely disabled patients [8–14]. A group of severe treatment-resistant OCD patients not submitted to DBS and treated with conventional therapy was not included in our study and would have constituted a good comparison group to ascertain changes over time in this kind of patients. However, a spontaneous remission or improvement of symptoms in this group of patients is extremely unlikely. The follow-up period in our study lasted just 12 months, so evidence of delayed adverse effects related to chronic brain stimulation as well as long-term sustainability of clinical response could not be assessed beyond this period. Conversely, patients may potentially reach responder status later than 12 months of follow-up. In addition, prior research with follow-up periods of several years does seem to indicate a sustained therapeutic response [42]. Finally, it cannot be ruled out that the participation of several centers may have influenced the variation found in some aspects of the therapy, such as electrode location (i.e. 56% of the active contacts were located in the AIC). This variation appeared even though all sites were trained on lead implant technique and were offered technical support (Supplementary Material). In this regard and considering the size of the electrode contacts, it must be said that the location of the geometric center of the active contact is only a reduced representation of the brain structures that are being stimulated. At the stimulation settings used in this study, contacts with a geometric center located in neighboring brain areas, e.g. the bed nucleus of the stria terminalis, may also reach the AIC, implying that more than 56% of hemispheres received stimulation in the AIC. In our opinion, our results reflect the current ‘real-world’ situation of DBS treatment in OCD, which is here prospectively assessed in an international multicentric way for the first time.

In conclusion, DBS for severe treatment-resistant OCD patients reduced the severity of obsessive-compulsive symptoms by an average of 40%, with a responder rate of 60%. Severe and permanent adverse effects happened, mainly related to infection and seizures. However, most AEs were mild or moderate, transient and related to stimulation and tended to resolve by adjustment of stimulation, as seen in DBS for movement disorders. In this severe treatment-resistant population, this open-label study supports that the potential benefits outweigh the potential risks of DBS.

## Supplementary information

Supplemental Material
