# Supplementary material for: A prospective international multi-center study on safety and efficacy of deep brain stimulation for resistant obsessive-compulsive disorder
Source: Mol Psychiatry. 2019 Oct 29;26(4):1234–47. doi: 10.1038/s41380-019-0562-6 (PMC7985042; doi:10.1038/s41380-019-0562-6)
Supplement: Supplementary file 1 — Supplemental Material [file 41380_2019_562_MOESM1_ESM.docx]

SUPPLEMENTARY MATERIAL

*Table of contents*

1. Supplementary Methods

1.1. Patient selection

1.2. Study design

1.2.1. Screening, Preoperative and Implant Phase

1.2.2. Evaluation of the electrode position

1.2.3. Parameter Selection and Treatment Phase

1.2.4. Methods to minimize potential bias

1.2.5. Investigator Training and Technical Support

1.3. Safety measurements

1.3.1. Definition and Classification of Adverse Events

1.3.2. Recording and Reporting of Adverse Events

1.3.3. Clinical Events Committee

1.3.4. Acute Stimulation-Induced Effects

1.4. Efficacy measurements

1.4.1. Yale-Brown Obsessive Compulsive Scale (Y-BOCS)

1.4.2. Global Assessment of Functioning (GAF)

1.4.3. Montgomery-Åsberg Depression Rating Scale (MADRS)

1.4.4. Young Mania Rating Scale (YMRS)

1.4.5. Clinician Global Impressions of Improvement (CGI-I) and Severity (CGI-S)

1.4.6. EuroQol group-5 Dimensional (EQ-5D)

1.5. Statistical analyses

2. Supplementary Results

2.1. Pharmacological treatment and psychotherapy

2.2. Final leads location

3. Supplementary Tables and Figures

3.1. Supplementary Table 1**.** Inclusion and exclusion criteria

3.2. Supplementary Table 2. Demographic and OCD clinical characteristics of enrolled patients

3.3. Supplementary Figure 1. Diagram of inclusion

3.4. Supplementary Figure 2. Study flow

3.5. Supplementary Figure 3. Study timeline

1. Supplementary Methods

1.1 Patient Selection

A study sample size of approximately 32 patients was planned to be enrolled across approximately 10 centers in Europe and Israel with a variety of expertise levels in DBS for OCD, from pioneers of the therapy to more recent adopters (3 to 5 patients per center). Inclusion and exclusion criteria are shown in Supplementary Table 1 and demographic data and OCD characteristics are summarized in Supplementary Table 2. Inclusion criteria were a principal diagnosis of OCD of disabling severity according to the structured clinical interview of the Diagnostic and Statistical Manual of Mental Disorders (DSM-IV-TR)^19^, with a Yale-Brown Obsessive-Compulsive Scale (Y-BOCS) score of at least 30/40. This level of impairment should have persisted for ≥ 5 years despite a minimum of three adequate trials (except in case of intolerance) with first- and/or second-line medications (at least 1 trial had to be with clomipramine) and a supplementary augmentation trial (atypical neuroleptics). Patients had not responded to an adequate trial of Cognitive Behavioral Therapy (CBT), consisting of ≥ 20 sessions of exposure and response prevention from a trained cognitive behavioral therapist. Patients had to be at least 18 years, not pregnant and using a medically acceptable method of contraception if female. They had to be able to understand and comply with instructions and provide their own written informed consent to be included in the study. Patients were excluded in case a) of current Axis I disorder that was primary to the OCD as demonstrated by the Structured Clinical Interview for DSM-IV-TR Axis I Disorders (SCID-I), b) of any clinically significant disorder or medical illness affecting brain function or structure (other than motor tics or Gilles de la Tourette syndrome), c) of a history of a neurosurgical ablation procedure in the target area, d) they previously received DBS therapy for any indication, e) they met DSM-IV-TR criteria for substance abuse or dependence ≤ 6 months prior to the screening session, f) they had a comorbid personality disorder that in the opinion of the investigator would have jeopardized the patient’s safety or study compliance, g) or a suicide attempt requiring medical treatment ≤ 3 months prior to the screening tests, or h) they posed a suicide risk in the opinion of the investigator (or an item 10 MADRS response ≥ 5). Other comorbid psychiatric diagnoses were not an exclusion criterion provided OCD was the primary diagnosis. Diagram of inclusion is shown in Supplementary Figure 1.

1.2 Study Design

This was a prospective, interventional, open-label, multi-center, post-market study aimed to confirm the safety and performance of Reclaim® DBS Therapy for OCD in adult patients with chronic and severe treatment-resistant OCD. Reclaim® DBS Therapy consists of bilateral electrical stimulation of the anterior internal capsule (AIC) as an adjunct to medication and as an alternative to anterior capsulotomy for treatment of chronic, severe, treatment-resistant OCD in adult patients who have failed at least 3 SSRIs and cognitive behavioral therapy. The primary objective of the study was to characterize adverse events (AEs) associated with the implantation procedure in the AIC, device and bilateral stimulation of this brain region in OCD patients. The second objective was to characterize treatment efficacy through Y-BOCS and additional measurements (GAF, MADRS, YMRS, CGI, EQ-5D).

The study comprised two main phases: a) a Screening, Preoperative, and Implant Phase, and b) a Parameter Selection and Treatment Phase. Unscheduled visits could be conducted as required for treatment of the patient’s OCD or other mental health problems between the scheduled visits. The total study duration was approximately 4 years and the total study duration per patient 14 months. Study flow (including recruitment procedures) is shown in Supplementary Figure 2.

1.2.1. Screening, Preoperative and Implant Phase

During the screening visit, medication and psychiatric history was documented, as well as OCD severity in order to determine study eligibility. AE data were collected and reported from the time Study Informed Consent was signed. The measures collected during the preoperative psychiatric visit served as the baseline data for analysis purposes. The DBS systems were implanted by the neurosurgeon within 6 weeks of the preoperative psychiatric visit. A pre-operative MRI was conducted to look at potential intracranial lesions that would discard the surgery. In some patients this MRI was also used for defining the lead location for the stereotactic implantation.

Model 3391 DBS leads [Medtronic, Inc.] were implanted bilaterally in the VC/VS region and connected subcutaneously to unilateral or bilateral Model 7428 Kinetra or bilateral Model 7426 Soletra Neurostimulators via Model 7482(A)/7483 Low Profile Extensions. The Soletra neurostimulator (Model 7426) accommodates one extension and lead, and therefore two neurostimulators had to be implanted for bilateral therapy. The Kinetra dual-channel neurostimulator (model 7428) accommodates one or two extensions and leads, and thus provides unilateral or bilateral neurostimulation from a single neurostimulator. The use of a commercialized image-guided software system was required for all lead implantations. Intraoperative stimulation (to verify correct placement of the electrodes) could be conducted in accordance with the intraoperative stimulation protocol detailed in the technical manual. To prevent flipping or migration of the neurostimulator, the suture holes in the connector block were used to secure the neurostimulator to the muscle fascia with 2-0 non-absorbable suture material. Post-operative imaging was performed to document lead location (CT and/or MRI were used according to the approved guidelines).

1.2.2. Evaluation of the electrode position

Patients were implanted with bilateral quadripolar leads, featuring 3mm-long contacts 4mm apart, spanning a total length of 24 mm (3391, Medtronic, Inc.), but often only one contact per hemisphere was actively used for stimulation. It is generally assumed that stimulation effects mainly take place at the negative contacts (cathodes), while clinically relevant effects near the positive anode(s) are negligible.^7, 40, 41^ To investigate in more detail which brain regions were actually being stimulated, we indicated the center of the cathodes on Mai’s brain atlas.^7, 42^ Using pre- and post-operative MR and CT scans merged with Medtronic FrameLinkTM Software (Medtronic, Inc.), we localized the cathodes for each patient at 12 months follow-up. All digital images were manually reformatted along the AC-PC plane. Contact positions were determined in consensus by four observers (LL, KL, FG & BN) and transferred to the atlas plates, while remaining blinded to the individual psychiatric outcome.

1.2.3. Parameter Selection and Treatment Phase

Following surgery recovery, patient-specific parameters for the programmable stimulation systems were established at the Parameter Selection visit, which occurred within 4 weeks of device implant. The goals were to identify optimal stimulation parameters (electrode configuration, amplitude, frequency, pulse width), and to ensure that the charge density did not exceed the safety limit of 30 microCoulombs/cm^2^/phase. The manufacturer-suggested stimulation parameters selection protocol provides a systematic evaluation of acute stimulation-induced effects for possible electrode configurations and parameter sets to identify those settings which provide a therapeutic effect without undesirable stimulation-induced effects. It was assumed that the setting that provided immediate (acute) therapeutic mood effects would also provide chronic relief of OCD symptoms.

Study visits were scheduled at 3, 6 and 12 months post-implant (study timeline is shown in Supplementary Figure 3). Patients were assessed for AE occurrence and treatment efficacy at each visit, measured by Y-BOCS, GAF, MADRS, YMRS, CGI and EQ-5D ratings as part of the psychiatric assessments (see below). Investigators could change stimulation parameters in response to AEs and/or to improve efficacy as necessary (in scheduled and unscheduled visits). Unscheduled visits were defined as visits to the investigator’s office required for the treatment of the patient’s OCD or other mental health problem occurring between the scheduled visits.

1.2.4. Methods to minimize potential bias

Methods incorporated to minimize potential bias included the following:

- Diverse geographical site representation with varied levels of expertise in DBS for OCD.

- Systematic identification of potential subjects via screening log

- Definition of inclusion/exclusion criteria as close as possible to the CE marked indication and Information for Prescribers documentation, to ensure a representative study population

- Clinical investigation plan allowing an as “real-world” follow-up schedule as possible, including unscheduled visits as needed.

- Design of the study in collaboration between sponsor and physicians (LG & BN)

- Standard procedures and data collection requirements with a common electronic database for all sites.

- Adjudication of device-related events by an independent Clinical Event Committee. The CEC was to consist of a minimum of 3 members. The CEC was responsible for the evaluation and categorization of AEs and the adjudication of etiology, procedure-relatedness, or device-relatedness including therapy-relatedness. The clinicians were to have specialties appropriate to the therapeutic area for which they reviewed AEs. Members were non-investigators. A minimum of one CEC member reviewed all AEs. The full CEC was to review all deaths, SAEs, SADEs, and ADEs. Medtronic personnel could facilitate the CEC meetings but could not be voting members.

- Assessment of lead location was performed by at least 2 blinded assessors (1 Medtronic expert and 1 independent neurosurgeon).

- Sponsor did not perform data collection.

- Interpretation of the data, writing of the report and decision to submit the paper for publication done in collaboration between the sponsor and the investigators, taking all parties inputs into account before reaching a consensus.

1.2.5. Investigator Training and Technical Support

A total of 4 investigator meetings were held, including training on the clinical investigation plan and study procedures. In addition, the following training procedures were performed during the course of the study:

• ECMT (European Continuing Medical Training) was provided in Leuven, Belgium, to each site at the beginning of the study and in 2012 following the third investigator meeting. The first ECMT provided a theoretical and practical training and the second one was focused on the practice (programming for the psychiatrist and surgery for the surgeons).

• On-site training for psychiatrists and neuropsychologists to assure the quality of testing and data.

Technical support during surgery was provided upon request from the study beginning until the third investigator meeting and was provided at each surgery from the third investigator meeting onward thereafter in order to ensure accuracy of the implant procedure.

1.3. Safety measurements

1.3.1. Definition and Classification of Adverse Events

Patients were assessed for Adverse Events (AEs) occurrence at each visit. After the patient had had an opportunity to spontaneously mention any problems, the investigator inquired about the occurrence of AEs via series of open-ended questions. All AEs were classified according to EN ISO14155:2011.

*Adverse Event*

An AE was defined as any untoward medical occurrence, unintended disease or injury, or untoward clinical signs in patients, users or other persons, whether or not related to the investigational medical device. This definition included any untoward medical event occurring after study enrollment even if no study treatment had been administered. Examples of an AE included (but were not limited to): any relevant abnormal laboratory finding, a new illness or injury unrelated to the device or therapy, worsening of psychiatric symptoms (e.g. anxiety, depression) that required an intervention other than reprogramming the device (such as an office visit, emergency room visit, or modification to a psychotropic medication), or a clinical observation temporally associated with the use of a device, whether or not considered related to the device. AEs did not include reprogramming of the DBS system due to lack of efficacy, transient undesirable stimulation-produced effects (see below), any normal expected postoperative complaints or symptoms (up to 30 days post-operative) if they did not require interventions differing from ordinary postoperative care, or any preexisting condition (unless there was a worsening of that condition in terms of nature, severity or frequency), among others.

*Serious Adverse Event*

A SAE was defined as any AE that led to death, fetal distress, fetal death or a congenital abnormality or birth defect, serious deterioration in the health of the patient that either resulted in: a) a life-threatening illness or injury, or b) a permanent impairment of a body structure or a body function, or c) in-patient or prolongation of hospitalization, or d) medical or surgical intervention to prevent life-threatening illness or injury or permanent impairment to body structure or a body function.

1.3.2. Recording and Reporting of Adverse Events

All AEs, regardless of relatedness or outcome, were collected throughout the study and reported on an electronic case report form (eCRF). Information reported on the AE included a description of the event, the date of AE onset (or the date the AE was first noticed by the investigator), the severity of the AE, the relatedness of the AE to the procedure, the device or the stimulation therapy, actions taken as a result of the AE and the outcome of the AE. Each unique event was documented separately. For those AEs determined to be related to stimulation therapy, the investigator reported the strength of the relatedness using the terms ‘definite’, ‘probable’ or ‘possible’.

All AEs and SAEs were followed up in accordance with good medical practice until resolved or judged no longer clinically significant. Supplemental measurements and/or evaluations that were necessary to fully investigate the nature and/or causality of an AE or SAE were also reported. When an AE was ongoing at 12 months follow-up visit, the patient exited through the regular study process and the AE was followed until resolution or stabilization.

AEs were coded using the Medical Dictionary for Regulatory Affairs (MedDRA) version 8.0. AEs were summarized according to the MedDRA High Level Group Terms (HLGT), which groups AEs that are related to each other by anatomy, pathology, physiology, etiology or function.

1.3.3. Clinical Events Committee (CEC)

An independent CEC reviewed all AEs. It consisted of 3 non-investigator clinicians. The CEC was responsible for the evaluation and categorization of AEs and the adjudication of etiology, procedure-relatedness, or device-relatedness including therapy-relatedness. The clinicians were to have specialties appropriate to the therapeutic area for which they reviewed AEs. Members were non-investigators. A minimum of one CEC member reviewed all AEs. The full CEC was to review all deaths, SAEs, SADEs, and ADEs. Medtronic personnel could facilitate the CEC meetings but could not be voting members.

1.3.4. Acute Stimulation-Induced Effects

ASIEs were transient, undesirable stimulation-induced effects that occurred during any programming session and that resolved with or without programming changes prior to the patient leaving a study follow-up visit (they did not require further follow-up or medical care outside the programming session). All study patients were monitored in the clinic for a minimum of 30 minutes for stimulation-induced effects after the final programming was set. Examples of ASIEs included (but were not limited to): somatosensory effects (e.g. paresthesia, sensations of cold or hot, cramping sensations), autonomic effects (e.g. facial flushing, tachycardia, hyperhidrosis), motor effects (e.g. muscular contractions), seizures, mood and anxiety effects (e.g. mood elevation, irritability, hypomania, increased anxiety or depressive symptoms).

1.4 Efficacy measurements

The main efficacy objective (the study’s secondary objective) was to compare Y-BOCS at 3, 6 and 12 months post-implant with pre-operative baseline. Other additional measurements were also performed, as part of the psychiatric assessment at the same visits (GAF, MADRS, YMRS, CGI and EQ-5D).

1.4.1. Yale-Brown Obsessive Compulsive Scale (Y-BOCS)

The Y-BOCS is a clinician-administered scale that consists of 10 individual questions, each with a scoring range of 0-4. Five of the questions relate to obsessions and five to compulsions. The total score can range from 0 (no symptoms) to 40 (most severe), and each subscale from 0 to 20. Y-BOCS responders were defined as having ≥ 35% improvement in the Y-BOCS total score from baseline.

1.4.2. Global Assessment of Functioning (GAF)

The GAF is a clinician-administered assessment tool designed to measure psychological, social and occupational functioning along a continuum from 0 (-health) to 100 (+health).

1.4.3. Montgomery-Åsberg Depression Rating Scale (MADRS)

The MADRS is a standardized and widely used, clinician-administered, 10-item scale that assesses the severity of depressive symptoms through direct observation. Items are scored from 0-6 with 6 representing the most severe symptoms. The scale’s total scores can range from 0 to 60, with scores ≥ 16 representing moderate-to-severe depression.

1.4.4. Young Mania Rating Scale (YMRS)

YMRS is a clinician-administered questionnaire to measure the severity of manic symptoms. The checklist consists of 11 items that assess such traits as mood, energy, irritability, speech, aggressive behavior, and appearance. Each item is scored on a 0 to 4 or a 0 to 8 scale. Total scores are from 0-60 with the higher score indicating more severe symptoms according to the following: ≤ 13: minimal; 13-20: mild; 21-26: moderate; and ≥ 38: severe.

1.4.5. Clinician Global Impressions of Improvement (CGI-I) and Severity (CGI-S)

The CGI scale is a standardized clinician-administered assessment tool designed to rate the severity of psychiatric illness, change in severity over time, and efficacy of medication. Two of the three global subscales were used in this study: Severity of Illness (CGI-S) and Global Improvement (CGI-I). The CGI-S assesses the clinician’s impression of the patient’s current illness state. Scores range from 1 (‘not ill’) to 7 (‘among the most extremely ill’). The CGI-I assesses the patient’s improvement or worsening from baseline. Scores range from 1 (‘very much improved’) to 7 (‘very much worse’).

1.4.6. EuroQol group-5 Dimensional (EQ-5D)

The EQ-5D is a standardized patient self-administered instrument for use as a measure of health outcome. It consists of a descriptive profile with 5 dimensions (mobility, self-care, usual activities, pain or discomfort, and anxiety or depression), in which each dimension has three different levels of severity (no problems, some problems, or extreme problems). A single index value for health status is calculated from the 5 dimensions. The index value ranges from 0 (worst) to 1 (best). The EQ-5D also includes a vertical visual analogue scale (EQ-VAS) that generates a self-rating of health-related quality of life ranging from ‘best imaginable health state’ to ‘worst imaginable health state’. The VAS ranges from 0 (worst) to 100 (best). The information can be used as a quantitative measure of health outcome.

1.5. Statistical analyses

The study was a prospective, interventional, open-label, single-arm, multi-center, post-market study. A representative sample of centers was chosen to provide a real-world post-market assessment of safety of the device and therapy.

A probability-based sample size was calculated to ensure enrollment of an adequate number of patients to attain a strong confidence of detecting common, clinically significant AEs. A minimum of 29 implanted patients was needed to provide greater than 95% probability of detecting at least one event, if the true event rate was 10% or greater. The resultant sample size of 30 implanted patients provided greater than 95% confidence of detecting events with rates of 10% or greater. In summary, the aim of the study was to enroll a sample large enough to give strong confidence that most common adverse events could be detected.

The results are presented as descriptive statistics for continuous variables. Mean and standard deviations are typically reported, with medians reported for non-normally distributed data. Proportions or rates of patients are reported for categorical variables. For rates of patients, the sample generally includes all 31 enrolled patients for the safety analyses, and the 30 implanted patients for the efficacy analyses.

There was no imputation of missing data for the safety or efficacy analyses, with the one exception of the Y-BOCS responder analysis, where patients with missing Y-BOCS were considered to be non-responders.

The study was not designed for performing significance testing.

2. Supplementary Results

2.1. Pharmacological treatment and psychotherapy

At the baseline visit, current CBT was the most frequent non-pharmacological therapy (9, 29%), followed by outpatient psychiatric care program (6, 19%). Regarding pharmacological treatment, clomipramine was the antidepressant most frequently taken at baseline (16, 52%), at a mean (SD) daily dose of 180 (93) mg, followed by escitalopram and paroxetine. Other psychotropic medications taken at baseline, either in monotherapy or along with an antidepressant, were antipsychotics in 20 patients (65%, most frequently quetiapine), anxiolytics in 15 patients (48%, most frequently alprazolam, clonazepam, and lorazepam) and hypnotics in 7 patients (23%, most frequently lormetazepam and zolpidem). The most frequently reported therapeutic classes of concomitant medications (excluding psychotropic medications) were drugs for peptic ulcer and gastro-esophageal reflux disease (6, 19%).

2.2. Final leads location

For contact 0, the BST was the most common location (37 leads, 62%), followed by the ventral part of the internal capsule (11, 18%), lateral hypothalamus (8, 13%), and fornix (4, 7%) (the location of active electrode cathodes at 12 months follow-up is shown in Figure 1).

3. Supplementary Tables and Figures

3.1. Supplementary Table 1**.** Inclusion and exclusion criteria

| INCLUSION CRITERIA |
| --- |
| To be eligible for inclusion into this study, subjects had to fulfill all of the following criteria prior to study enrollment: |
| - Subject has signed and dated the ICFs - Subject meets DSM-IV-TR (Diagnostic and Statistical Manual of Mental Disorders, Fourth Edition – Text Revision) diagnostic criteria for OCD - Subject has medical record documentation of an OCD diagnosis of disabling nature ≥5 years - Subject has a Y-BOCS score ≥30 - Subject’s OCD is chronic and treatment-resistant, defined as:   - Subject has not clinically responded to a minimum of 3 documented treatments of adequate dose and duration with first- and/or second- line medications (at least 1 treatment must have been with clomipramine) and a supplementary augmentation treatment     - First-line medications: SSRIs / venlafaxine     - Second-line medications: clomipramine     - Augmentation strategy: atypical neuroleptics     - Adequate treatment: more than 3 months at an adequate dose (at or if tolerated, beyond the maximum recommended dose)   - Subject has not clinically responded to an adequate treatment of CBT or has demonstrated marked intolerance to CBT (in therapist’s judgment)     - Adequate treatment: ≥20 sessions of exposure and response prevention (ERP) from a trained cognitive behavioral therapist - Subject is ≥18 years of age   Female subject, if of child-bearing potential, is not pregnant and is using a medically acceptable method of contraception. |
| EXCLUSION CRITERIA |
| Subjects who met any of the following criteria were not included in the study: |
| - Subject has a current Axis I disorder that is primary to the OCD as demonstrated by the Structured Clinical Interview for DSM-IV-TR Axis I Disorders (SCID-I)^^[[1]](#footnote-2)^^ - Subject has a neurological condition that may jeopardize the subject’s ability to give informed consent, follow study requirements, or that may confound the subject’s diagnosis or assessments - Subject has any medical contraindications to undergoing implantation of a bilateral Reclaim^®^ DBS system - Subject has a history of a neurosurgical ablation procedure in the target area - Subject has previously received or is currently receiving DBS therapy for any indication - Subject meets DSM-IV-TR criteria for substance abuse or dependence ≤6 months prior to the screening tests - Subject is determined during the clinical interview to have a co-morbid personality disorder that in the opinion of the investigator may jeopardize the subject’s safety or study compliance - Subject made a suicide attempt requiring medical treatment ≤3 months prior to the screening tests - Subject has a history of 2 or more suicide attempts ≤12 months prior to the screening tests - Subject poses a serious suicide risk as indicated by any of the following:   - Serious plans for suicide as identified by a MADRS item 10 response of ≥“5”   - The opinion of the investigator - Subject is currently enrolled in or plans to enroll in any concurrent drug and/or device study that may confound the results of this study as determined by Medtronic - In the opinion of the investigator, patient is expected to be non-compliant to follow-ups or attendance of study visits. |

^1^ The SCID-I is a clinician-administered, semi-structured interview with 9 diagnostic modules used to assess a variety of psychiatric diagnoses in accordance with DSM-IV-TR. The interview includes both required probe questions and suggested follow-up questions, with numerous skip-out directions, where applicable, to derive a diagnosis

3.2. Supplementary Table 2. Demographic and OCD clinical characteristics of enrolled patients

| Characteristic | Mean | Standard Deviation | Range |
| --- | --- | --- | --- |
| Gender (% male) | 48% |  |  |
| Age (years) | 41.0 | 9.9 | 22-60 |
| Education (% graduated high school) | 65% |  |  |
| Employment Status (% currently working) | 10% |  |  |
| Baseline Y-BOCS | 34.7 | 2.9 | 30-40 |
| Age at Onset of OCD | 16.5 | 7.9 | 7-44 |
| Age at Onset of Disabling OCD | 25.5 | 9.0 | 13-46 |
| Years Since Onset of OCD | 24.5 | 9.0 | 8-42 |
| Years Since Onset of Disabling OCD | 15.6 | 8.3 | 5-42 |
| Lifetime Failed Medication Trials | 10.0 | 3.5 | 5-21 |
| Prior Suicide Attempt (%) | 26% |  |  |
| Current Cognitive Behavior Therapy (%) | 29% |  |  |

Total N= 31 enrolled patients

Y-BOCS: Yale-Brown Obsessive Compulsive Scale; OCD: Obsessive-Compulsive Disorder

3.3. Supplementary Figure 1. Diagram of inclusion

32 screened

Screened

31 enrolled

30 implanted

30 initiated stimulation

1 discontinued after Month 3

1 explanted after Month 3 but continued in study

1 discontinued before implant

29 completed study

28 with implanted system

First Patient First Visit was on 30-Apr-2010, Last Patient Last Visit was on 02-Sep-2014

3.4. Supplementary Figure 2. Study flow

3.5. Supplementary Figure 3. Study timeline

1. [↑](#footnote-ref-2)
